# Impact of parental oral health literacy on orthodontic treatment need in schoolchildren

**DOI:** 10.1590/2177-6709.30.2.e2524238.oar

**Published:** 2025-05-23

**Authors:** Gélica Lima GRANJA, Larissa Chaves Morais de LIMA, Tiago Ribeiro LEAL, Veruska Medeiros Martins BERNARDINO, Érick Tássio Barbosa NEVES, Saul Martins PAIVA, Ana Flávia GRANVILLE-GARCIA

**Affiliations:** 1 State University of Paraíba, Dental School, Department of Dentistry (Campina Grande/PB, Brazil).; 2 Federal University of Minas Gerais, Dental School, Department of Paediatric Dentistry and Orthodontic (Belo Horizonte/MG, Brazil).

**Keywords:** Malocclusion, Orthodontic treatment need, Health literacy, Má oclusão, Necessidade de tratamento ortodôntico, Letramento em saúde

## Abstract

**Introduction:**

Malocclusion is a global health problem and when not treated in childhood, can persist throughout one’s lifetime.

**Objective:**

As children are dependent on their parents/caregivers for health care, the aim of the present study was to investigate the association between parental oral health literacy and orthodontic treatment need in schoolchildren.

**Methods:**

A descriptive, analytical, cross-sectional study with a representative sample of children eight to ten years of age was conducted. Sociodemographic questionnaire, the Sleep Disturbance Scale for Children and the Oral Health Literacy - Adult Questionnaire were sent for parents/caregivers. Children answered the Revised Children’s Manifest Anxiety Scale. For the diagnosis of orthodontic treatment need, the Dental Aesthetic Index was used. Data analysis included multinomial logistic regression analyses (OR) (p < 0.05).

**Results:**

Treatment need was identified for 55.2% of participants. Children with anxiety were 53% more likely to have mandatory treatment needs (OR = 1.53; 95% CI: 1.16-2.57; p = 0.04), children with sleep disturbances were 1.94 times more likely to have mandatory treatment needs (OR = 1.94; 95% CI: 1.29-2.91; p = 0.01). Children whose parents/caregivers had an insufficient level of oral health literacy were 77% more likely to have mandatory treatment needs (OR = 1.77; 95% CI: 1.04-2.99; p = 0.03).

**Conclusions:**

Orthodontic treatment need was greater among schoolchildren from families with a lower income, who lived in homes with more than five residents, whose mothers were younger, whose parents/caregivers had an insufficient level of oral health literacy, and those with sleep disorders and with anxiety.

## RESUMO

**Introdução:**

A má oclusão é um problema de saúde global e, quando não tratada na infância, pode persistir por toda a vida.

**Objetivo:**

Como as crianças são dependentes dos pais/responsáveis para cuidados de saúde, o objetivo do presente estudo foi investigar a associação entre o letramento parental em saúde bucal e a necessidade de tratamento ortodôntico em escolares.

**Material e Métodos:**

Estudo transversal, descritivo e analítico, com amostra representativa de crianças com 8 a 10 anos. Um questionário sociodemográfico, a *Sleep Disturbance Scale for Children* (SDSC) e o *Oral Health Literacy-Adult Questionnaire* (OHL-AQ) foram enviados para os pais/responsáveis responderem. As crianças responderam à *Revised Children’s Manifest Anxiety Scale* (RCMAS). Para o diagnóstico da necessidade de tratamento ortodôntico, foi utilizando o Índice Estético Dentário (DAI). A análise dos dados envolveu a análise de Regressão Logística multinomial (OR) (*p*< 0,05).

**Resultados:**

A necessidade de tratamento foi identificada em 55,2%. Crianças com ansiedade tiveram 53% mais chances de ter necessidade de tratamento obrigatório (OR = 1,53; IC 95%: 1,16-2,57; *p*= 0,04), crianças com distúrbios do sono tiveram 1,94 vezes mais chances de ter necessidade de tratamento ortodôntico obrigatório (OR = 1,94; IC 95%: 1,29-2,91; *p*= 0,01). Crianças cujos pais/responsáveis tinham um nível insuficiente de letramento em saúde bucal tiveram 77% mais chances de ter necessidade de tratamento obrigatório (OR = 1,77; IC 95%: 1,04-2,99; *p*= 0,03).

**Conclusões:**

A necessidade de tratamento ortodôntico em crianças na fase de dentição mista foi associada à baixa renda familiar, residir com mais de cinco moradores, filho de mãe com até 40 anos de idade, cujos pais/cuidadores têm conhecimento insuficiente sobre saúde bucal; assim como apresentar distúrbios do sono e ansiedade.

## INTRODUCTION

Malocclusion is a global public health problem and is considered the third most common oral health problem after dental caries and periodontal disease.^1^ ^-^ ^3^ Besides compromising chewing function and aesthetics, malocclusion can exert a negative impact on self-esteem, social interactions and quality of life.^4^ ^-^ ^8^ When not treated in childhood, the severity of malocclusion tends to increase with age and can persist throughout one’s lifetime.^9^ ^,^ ^10^

Treatment for this dental problem should be performed as early as possible.^9^ However, the choice of seeking treatment on the part of the patient is conditioned by the perceived impact of malocclusion on oral health and aesthetics.^11^ By the mixed dentition phase, children are aware of their appearance and have already acquired a sense of aesthetics,^12^ which underscores the importance of treatment in this phase, as children have not yet experienced the peak growth spurt, favoring the prognosis of the treatment of skeletal malocclusions.^13^

Studies conducted with children and adolescents have identified that the need for orthodontic treatment is high, ranging from 30.9% to 85.9%.^14^ ^,^ ^15^ As children are dependent on their parents/caregivers for health care,^16^ offering parents/caregivers more comprehensive counseling on the consequences of malocclusion and stressing the importance of treatment can play a crucial role in the reduction in the prevalence of this health problem.^12^ ^,^ ^17^

As children depend on their caregivers, the family context exerts a substantial influence on their oral health.^18^ Thus, knowledge on factors related to caregivers is fundamental. The oral health literacy (OHL) of parents/caregivers has attracted the attention of researchers, as this factor can exert an impact on oral health outcomes in children. OHL is the capacity to acquire, process and understand basic oral health information with regards to prevention, treatment and dental services, as well as the application of such knowledge to modify behaviors.^19^ ^,^ ^20^ Studies have found associations between low parental OHL and dental caries in children.^21^ ^-^ ^25^ Despite the relevance, no studies were found that investigated the association between parental OHL and malocclusion in children. 

Analyses involving the family context can contribute to a more comprehensive understanding of factors that influence the seeking of orthodontic treatment for children. Besides providing information that can promote reflections on the importance of ensuring access to treatment for malocclusions, especially for the portion of the population in situations of social vulnerability, epidemiological data of this type can assist in the allocation of health resources. The conceptual hypothesis of the present investigation is that parents/caregivers with low OHL fail to seek orthodontic treatment for their children. 

Therefore, the aim of the present study was to investigate the association between the oral health literacy of parents/caregivers and orthodontic treatment need in children eight to ten years of age through multinomial analysis.

## MATERIAL AND METHODS

The present study was conducted in accordance with the ethical precepts stipulated in the Declaration of Helsinki and received approval from the local institutional review board (certificate number: 10514619.2.0000.5187). The parents/caregivers of the patients received explanations with regards to the objectives of the study and signed a statement of informed consent, authorizing the participation of their children. Children who agreed to participate signed a consent form. 

## STUDY DESIGN AND SAMPLE

This study was reported following the guidelines of the Strengthening the Reporting of Observational Studies in Epidemiology (STROBE statement). A descriptive, analytical, cross-sectional study was conducted with a representative sample of Brazilian children, eight to ten years of age, at 73 public and 58 private schools.

The sample size was calculated for analytical studies of comparisons between two independent proportions using the G*Power software program, version 3.1 (Franz Faul, Universitat Kiel, Germany), considering a 95% significance level and 80% test power. The proportions of malocclusion between children with and without sleep disturbance were estimated to be 54.1% and 40%, respectively, based on the pilot study. Although it was not the primary independent variable, the sleep disturbance variable was the only one that provided the highest prevalence and, consequently, the largest sample size to investigate the associations of interest in this study. A complex sampling method was employed, with the random selection of schools followed by the selection of students using a simple randomization method. Schools from each of the six administrative districts of the city were included proportionally to their representativeness in terms of the number of students eight to ten years of age. The minimum sample was calculated to be 393 students, to which a design effect of 1.6 was applied, reaching a sample of 628 students. Twenty percent was then added to compensate for possible losses, determining a target sample of 785 individuals.

## INCLUSION AND EXCLUSION CRITERIA

Children of both sexes eight to ten years of age enrolled at public and private schools were included. Children with a present or past history of orthodontic treatment, those with syndromes, developmental disorders or cognitive deficit (reported by the teachers) were excluded from the study.

## TRAINING AND CALIBRATION EXERCISES

An examiner with expertise in Orthodontics led the training of four dentists for the diagnosis of orthodontic treatment need using the Dental Aesthetic Index (DAI).^26^ The criteria for the diagnosis were initially analyzed using photographs, plaster models, clinical charts and the calculation of the DAI. Next, clinical examinations were performed on 40 children (20 from a public school and 20 from a private school selected by convenience). Inter-examiner agreement was determined by comparing the results of each examiner to those of the experienced orthodontist. After a seven-day interval, the four examiners repeated the examinations, to determine the intra-examiner agreement. These determinations were made using Cohen’s Kappa statistic, and the results revealed a good level of agreement (K > 0.81).

## PILOT STUDY

A pilot study was conducted with 40 children enrolled at two schools (one public and one private). The schools were selected by convenience and were excluded from the main study. 

## NON-CLINICAL DATA COLLECTION

A questionnaire addressing sociodemographic characteristics, the Sleep Disturbance Scale for Children (SDSC) and the Oral Health Literacy-Adult Questionnaire (OHL-AQ) were sent to the parents/caregivers to answer, for the collection of non-clinical data. The sociodemographic questionnaire addressed the child’s age, race and sex, mother’s age and schooling, family income and number of residents in the home. 

The SDSC is used to assess sleep disturbances in children and has been validated for use in Brazil. The instrument is composed of 26 items addressing sleep habits in the previous six months: difficulties in falling asleep and staying asleep, sleep-disordered breathing, arousal disorders, sleep-wake transition disorders, excessive daytime sleepiness and excessive sweating during sleep (hyperhidrosis). The response options are scored on a scale of 1 to 5: “never”, “occasionally” (once or twice per month), “sometimes” (once or twice per week), “often” (three to five time per week) and “always” (daily). The sum of the item scores gives the total score, which ranges from 26 to 130 points. In this study, a cutoff point of 39 was considered to characterize the presence of sleep disturbance in the children.^27^

The OHL-AQ is a self-administered questionnaire for the assessment of oral health literacy in adults that has been translated and validated for use in Brazil. The OHL-AQ is composed of 17 items distributed among four sections: reading comprehension, numeracy, listening comprehension and decision making. Each correct answer is attributed one point and incorrect answers receive zero points. The total ranges from 0 to 17 points, with higher scores denoting a more advance level of oral health literacy.^28^

The children answered the Revised Children’s Manifest Anxiety Scale (RCMAS), which is a psychometric measure for the investigation of anxiety in children 8 to 13 years of age. The RCMAS comprises 37 items divided into two subscales: an anxiety scale and “lie” scale. The score ranges from 0 to 37 points, with higher scores denoting a greater anxiety trait. For the present study, only the items addressing anxiety were used, corresponding to Items 1 to 28. The score was categorized in tertiles, resulting in a classification of high, medium and low levels of anxiety.^29^

## CLINICAL DATA COLLECTION

Four dentists first gave orientations on oral hygiene and supervised the children brushing their teeth. The clinical examinations were conducted with the child sitting in front of the examiner, who wore personal protective equipment and an LED lamp on the head (Petzl Zoom; Petzl America; Clearfield, UT, USA). Examinations were performed with a sterilized mouth mirror (PRISMA, São Paulo, Brazil), sterilized Williams probe (WHO-621; Trindade, Campo Mourão, Brazil) and gauze to dry the teeth, following the method recommended by the World Health Organization (WHO).^30^

The orthodontic treatment need was determined using the DAI, which is a quantitative index proposed by Cons et al.^26^ and recommended by the WHO^31^ for the assessment of the psychosocial impact of malocclusion, based on occlusal characteristics: missing teeth, anterior crowding, anterior spacing, midline diastema, greater maxillary anterior misalignment, greater mandibular anterior misalignment, maxillary horizontal overjet, anterior open bite and anteroposterior molar relationship.^26^

Considering the physiological characteristics of occlusal development during the mixed dentition phase, particularly in the “ugly duckling stage,” some adjustments were made to the components: visible missing teeth, midline diastema, greater anterior irregularity in the maxilla and mandible, anterior open bite, and molar anteroposterior relationship.^32^

The score enables the classification of the degree of malocclusion severity and orthodontic treatment need: DAI ≤ 25, normal occlusion, no treatment need; DAI = 26-30, definite malocclusion, elective treatment need; DAI = 31-35, severe malocclusion, treatment highly recommended; DAI ≥ 36, very severe malocclusion, mandatory treatment. 

## STATISTICAL ANALYSIS

Descriptive analysis was performed for all data, followed by unadjusted and adjusted multinomial logistic regression analyses with the calculation of odds ratios (OR) and respective 95% confidence intervals (CI), considering orthodontic treatment need (DAI score) as the dependent variable. Variables with a p-value < 0.20 in the unadjusted analysis were incorporated into the adjusted regression analysis, and those that enabled a better fit, remained in the final model: mother’s age, number of residents in the home, monthly family income, presence of childhood anxiety, presence of sleep disturbance and parental oral health literacy. The variables used to control for confounding factors were those with a p-value ≤0.20 in the unadjusted analysis.

In this study, the outcome variable was categorized according to the DAI instrument: no need for orthodontic treatment (≤25 points, normal occlusion); elective treatment need (26-30 points, defined malocclusion); mandatory treatment need (≥31 points, severe or very severe malocclusion).^26^

## RESULTS

The final sample consisted of 739 children aged 8 to 10 years, with 351 from public schools and 388 from private schools. The response rate was 94.1%, and losses occurred due to three consecutive absences by the children.

The characteristics of the sample are displayed in Table 1. Orthodontic treatment need was identified in 55.2%, with 22.7% classified as having elective treatment need and 32.5% classified as having mandatory treatment need. A large portion of the participants had anxiety (35.0%) and sleep disturbances (58.9%). The predominant classification of OHL among the parents/caregivers was marginal (36.3%).

**Table 1:** Characterization of sample.

Variables	n (%)
Sex	
Male	370 (50.1)
Female	369 (49.9)
Mother’s schooling	
≤ 8 years of study	310 (42.2)
> 8 years of study	425 (57.8)
Mother’s age	
≤ 40 years	574 (78.7)
> 40 years	155 (21.3)
Number of residents in home	
> 5	96 (13.2)
≤ 5	634 (86.8)
Monthly family income	
≤ R$ 1000	327 (57.0)
> R$ 1000	247 (43.0)
Orthodontic treatment need	
None	331 (44.8)
Elective	168 (22.7)
Mandatory	240 (32.5)
Childhood anxiety	
High	353 (34.2)
Medium	259 (35.0)
Low	227 (30.7)
Sleep disturbance	
Yes	435 (58.9)
No	304 (41.1)
Parental oral health literacy	
Insufficient	259 (35.0)
Marginal	268 (36.3)
Adequate	212 (28.7)
School type	
Public	351 (47.5)
Private	388 (52.5)

Table 2 displays the associations between the independent variables and orthodontic treatment need. With regards to sociodemographic variables, the adjusted regression model revealed that children whose mothers were up to 40 years of age were 71% more likely to have mandatory orthodontic treatment need, compared to no treatment need (OR = 1.71; 95% CI: 1.47-1.96; p = 0.04). The need for elective orthodontic treatment was 1.52 times greater among children who lived in a home with more than five residents (OR = 1.52; 95% CI: 1.28-1.96; p = 0.03). Children in families with a monthly income of up to US$ 172 were 1.81 times more likely to have mandatory treatment need (OR = 1.81; 95% CI: 1.03-3.19; p = 0.03).

**Table 2:** Distribution of sample according to orthodontic treatment need.

Variable	Orthodontic treatment need		
	No treatment need	Elective treatment	Mandatory treatment
	n (%)	n (%)	n (%)
Sex			
Male	171 (46.2)	74 (20.0)	125 (33.8)
Female	160 (43.4)	94 (25.5)	115 (31.2)
Mother’s schooling			
≤ 8 years of study	133 (42.9)	75 (24.2)	102 (32.9)
> 8 years of study	197 (46.4)	92 (21.6)	136 (32.0)
Mother’s age			
≤ 40 years	185 (48.2)	76 (19.8)	123 (32.0)
> 40 years	141 (40.9)	90 (26.1)	114 (33.0)
Number of residents in home			
> 5	286 (45.1)	140 (22.1)	208 (32.8)
≤ 5	40 (41.7)	28 (29.2)	28 (29.2)
Monthly family income			
≤ R$ 1000	171 (42.1)	89 (21.9)	146 (36.0)
> R$ 1000	81 (48.2)	43 (25.6)	44 (26.2)
Childhood anxiety			
High	103 (40.7)	64 (25.3)	86 (34.0)
Medium	127 (49.0)	48 (18.5)	84 (32.4)
Low	100 (44.5)	56 (24.7)	70 (30.8)
Sleep disturbance			
Yes	172 (39.5)	106 (24.4)	157 (36.1)
No	159 (52.3)	62 (20.4)	83 (27.3)
Parental oral health literacy			
Insufficient	111 (42.9)	50 (19.3)	98 (37.8)
Marginal	123 (45.9)	71 (26.5)	74 (27.6)
Adequate	97 (45.8)	47 (22.2)	68 (32.1)
School type			
Public	154 (43.9)	79 (22.5)	118 (33.6)
Private	177 (45.6)	89 (22.9)	122 (31.4)

With regards to individual variables, children with anxiety were 53% more likely to have mandatory treatment need (OR = 1.53; 95% CI: 1.16-2.57; p = 0.04) and children with sleep disturbances were 1.94 times more likely to have mandatory treatment need (OR = 1.94; 95% CI: 1.29-2.91; p = 0.01). Moreover, children whose parents/caregivers had an insufficient level of OHL were 77% more likely to have mandatory treatment need (OR = 1.77; 95% CI: 1.04-2.99; p = 0.03), compared to no treatment need. Furthermore, the regression analysis did not show a significant association between school type and the need for orthodontic treatment (Table 3).

**Table 3:** Multinomial logistic regression of orthodontic treatment need associated with sociodemographic factors, childhood anxiety, sleep disturbance and parental oral health literacy.

Variable	No treatment need				Elective treatment need				Mandatory treatment need			
	Unadjusted OR*		Adjusted OR**		Unadjusted OR*		Adjusted OR**		Unadjusted OR*		Adjusted OR**	
	p-value	(95% CI)	p-value	(95% CI)	p-value	(95% CI)	p-value	(95% CI)	p-value	(95% CI)	p-value	(95% CI)
Sex												
Male	-	-	-	-	0.90	0.98 (0.75 - 1.28)	-	-	0.02*	0.80 (0.66 - 0.97)	-	-
Female		-		-	-	-	-	-	-	-	-	-
Mother’s schooling												
≤ 8 years of study	-	-	-	-	0.47	1.10 (0.84 - 1.44)	-	-	0.08*	0.84 (0.70 - 1.02)	-	-
> 8 years of study	-	-	-	-	-	-	-	-	-	-	-	-
Mother’s age												
≤ 40 years	-	-	-	-	0.05*	1.92 (0.67 - 1.96)	0.03*	1.65 (1.03 - 1.54)	0.01*	1.95 (1.47 - 2.59)	0.04*	1.71 (1.47 - 1.96)
> 40 years	-	-	-	-	-	-	-	-	-	-	-	-
Number of residents in home												
> 5	-	-	-	-	0.01*	2.07 (1.46 - 2.93)	0.03*	1.52 (1.28 - 1.96)	0.95	0.99 (0.98 - 1.43)	-	-
≤ 5	-	-	-	-	-	-	-	-	-	-	-	-
Monthly family income												
≤ R$ 1000	-	-	-	-	0.54	1.09 (0.80 - 1.49)	-	-	0.01*	2.13 (1.66 - 2.72)	0.03*	1.81 (1.03 - 3.19)
> R$ 1000	-	-	-	-	-	-	-	-	-	-	-	-
Childhood anxiety												
Yes	-	-	-	-	0.37	1.15 (0.83 - 1.59)	-	-	0.01*	2.12 (1.65 - 2.52)	0.04*	1.53 (1.16 - 2.57)
No	-	-	-	-	-	-	-	-	-	-	-	-
Sleep disturbance												
Yes	-	-	-	-	0.01*	1.60 (1.21 - 2.12)	0.17*	1.52 (1.10 - 2.70)	0.01*	2.78 (2.19 - 3.52)	0.01*	1.94 (1.29 - 2.91)
No	-	-	-	-	-	-	-	-	-	-	-	-
Parental oral health literacy												
Insufficient	-	-	-	-	0.30	1.20 (0.84 - 1.70)	-	-	0.01*	2.14 (1.64 - 2.79)	0.03*	1.77 (1.04 - 2.99)
Marginal	-	-	-	-	0.61	0.91 (0.66 - 1.27)	-	-	0.17*	0.83 (0.64 - 1.08)	0.81	1.06 (0.63 - 1.77)
Adequate	-	-	-	-	-	-	-	-	-	-	-	-
School type												
Public	-	-	-	-	0.31	1.37 (0.87-1.17)	-	-	0.37	1.13 (0.78-1.55)	0.45	1.12 (1.02-2.07)
Private	-	-	-	-	0.08	1.78 (0.97-2.17)	-	-	0.12	1.32 (0.98-1.67)	0.09	1.76 (0.97-2.77)

## DISCUSSION

The results of the present study revealed that children from low-income families, those living in homes with more than five residents, those whose mothers were up to 40 years of age, those whose parents/caregivers had an insufficient level of OHL, those with anxiety and those with sleep disturbances were more likely to have orthodontic treatment needs. To the best of our knowledge, this is the first representative population-based study to investigate associations between these variables and orthodontic treatment need in children eight to ten years of age.

During the mixed dentition phase, significant dentoskeletal changes occur,^33^ a period during which children frequently engage in harmful oral habits that can lead to malocclusion.^34^ Furthermore, this phase is crucial for early orthodontic intervention, improving the prognosis of skeletal malocclusion treatment.^35^ ^,^ ^36^ Therefore, it is essential to investigate the factors associated with orthodontic treatment needs in this age group.

Orthodontic treatment needs are common among Brazilian children, possibly due to the unavailability of this type of service in the public healthcare system. Considering social issues in Brazil, such as inequalities in the distribution of income and employment, treatment to correct malocclusion is inaccessible for many families in situations of social vulnerability. This scenario is similar to other countries with significant income inequality, including Saudi Arabia and India.^37^ ^,^ ^38^ In contrast, orthodontic treatment for children and adolescents in developed nations, such as Austria and Norway, is subsidized by the government and distributed equitably irrespective of family income.^11^ ^,^ ^39^ Indeed, approximately one-third of Austrian children in the mixed dentition phase have access to orthodontic treatment subsidized by social security.^11^ If this model were adjusted to the Brazilian context, approximately nine million Brazilian children would have access to treatment for the correction of malocclusions.^40^ Although Brazil has a very extensive public healthcare system that already offers accessible dental care, compared to many countries, the implementation of preventive and interceptive orthodontic treatment remains incipient. Thus, adapting successful international public policy models to the Brazilian context could expand access to orthodontic treatment for millions of children.

The association found in this study between factors related to the mother and orthodontic treatment need may be explained by the influence of mothers on the oral health of their children. However, no significant association was found between maternal education level and the need for orthodontic treatment. Nevertheless, the literature suggests that factors such as low maternal education, low family income, inadequate oral hygiene habits, and lack of parental awareness regarding oral health care are risk factors for dental caries and dental trauma in childhood.^41^ ^,^ ^42^ These conditions may lead to tooth loss and, consequently, malocclusion.^43^ Furthermore, the occurrence of deleterious oral habits in childhood (such as pacifier use and thumb-sucking) depends on parents’/caregivers’ perceptions of the impact of these habits on the development of malocclusions. Therefore, although this study did not identify a statistically significant association, these factors are relevant for planning oral health policies.

An association between sleep disturbances and orthodontic treatment need was found in the present study. Malocclusions such as anterior open bite and posterior crossbite are risk factors for the development of sleep disturbances in children.^44^ Thus, early orthodontic treatment may play a crucial role in the reduction of the impacts of sleep disturbances in children, as inadequate sleep can have adverse effects on child development in the form of delayed growth, as well as cognitive and motor dysfunctions.^45^

Anxiety is one of the most prevalent psychological problems in childhood (19.6%) and is characterized by persistent fear in social situations involving exposure to the unknown or embarrassment.^46^ ^-^ ^49^ This disorder can trigger harmful oral habits and, consequently, the development of malocclusion,^47^ ^,^ ^50^ which may explain the greater orthodontic treatment need among anxious children found in the present study. 

An insufficient level of OHL on the part of parents/caregivers was associated with mandatory orthodontic treatment need in the children. Previous studies have also found an association between inadequate OHL and other oral health outcomes, such as dental caries.^22^ ^-^ ^25^ Understanding counseling and explanations given by the dentist is fundamental to the maintenance of oral health, and enables the early seeking of orthodontic treatment for the correction of malocclusion. 

This study has the limitations inherent to the cross-sectional design, which does not enable establishing cause-and-effect relationships between the variables studied. Furthermore, the use of self-reported data from parents/caregivers may incur in recall bias or a desire to respond in a socially acceptable manner. However, we also employed rigorous methods to minimize the risk of bias, such as the sample size calculation with the correction for the design effect to ensure representativeness, the execution of a pilot study to test the methods, the calibration of the examiners and the use of a validated instrument for measuring orthodontic treatment need.

The results of this study can be used in the planning of public policies directed at the implementation of orthodontic treatment in the public healthcare system, to reduce the impact of malocclusion on the quality of life of children. Moreover, measures should be directed at the training of dentists in the use of a screening tool, such as the DAI, to determine orthodontic treatment need. This study provides unprecedented data on the association between the oral health literacy of parents/caregivers and orthodontic treatment need in children. These data can assist in guiding longitudinal studies for the determination of the cause-and-effect relationship of these variables. 

## CONCLUSION

The need for orthodontic treatment in children during the mixed dentition phase was associated with low family income, living with more than five household members, having a mother under 40 years of age, and parents/caregivers with insufficient oral health knowledge. Additionally, sleep disturbances and anxiety were also associated with the need of orthodontic treatment.
